# Associations between disease severity, coping and dimensions of health-related quality of life in patients admitted for elective coronary angiography – a cross sectional study

**DOI:** 10.1186/1477-7525-6-38

**Published:** 2008-05-29

**Authors:** Bjørg Ulvik, Ottar Nygård, Berit R Hanestad, Tore Wentzel-Larsen, Astrid K Wahl

**Affiliations:** 1 Faculty of Health and Social Sciences, Bergen University College, Bergen, Norway; 2 Institute of Medicine, University of Bergen, Norway; 3 Department of Heart Disease, Haukeland University Hospital, Bergen, Norway; 4 Department of Public Health and Primary Health Care, University of Bergen, Norway; 5 Centre for Clinical Research, Haukeland University Hospital, Bergen, Norway; 6 Institute of Nursing and Health Sciences, Medical Faculty the University of Oslo, Oslo, Norway

## Abstract

**Background:**

In patients with suspected coronary artery disease (CAD), the overall aim was to analyse the relationships between disease severity and both mental and physical dimensions of health related quality of life (HRQOL) using a modified version of the Wilson and Cleary model.

**Methods:**

Using a cross-sectional design, 753 patients (74% men), mean age 62 years, referred for elective cardiac catheterisation were included. The measures included 1) physiological factors 2) symptoms (disease severity, self-reported symptoms, anxiety and depression 3) self-reported functional status, 4) coping, 5) perceived disease burden, 6) general health perception and 7) overall quality of life. To analyse relationships, we performed linear and ordinal logistic regressions.

**Results:**

CAD and left ventricular ejection fraction (LVEF) were significantly associated with symptoms of angina pectoris and dyspnea. CAD was not related to symptoms of anxiety and depression, but less depression was found in patients with low LVEF. Angina pectoris and dyspnea were both associated with impaired physical function, and dyspnea was also negatively related to social function. Overall, less perceived burden and better overall QOL were observed in patients using more confronting coping strategy.

**Conclusion:**

The present study demonstrated that data from cardiac patients to a large extent support the suggested model by Wilson and Cleary.

## Background

Symptoms related to Coronary Artery Disease (CAD) may have a major impact on mood, functional status, general health, dimensions of health-related quality of life (HRQOL) and overall quality of life [1-4]. Although there is a general agreement that HRQOL is a multidimensional construct [5-8], the associations between the dimensions in HRQOL lack a solid theoretical framework [9,10]. Among few conceptual models, Wilson and Cleary [5] highlights certain relationships between different dimensions of HRQOL. This model indicates that biological and physiological processes affect the perception of symptoms, which in turn affects functioning, general health perception and overall QOL. However, they point out that the main causal direction in their model does not imply that there are not reciprocal relationships [5].

With regard to previous research, weak associations have been found between objective measures of disease, symptoms, function and well-being in different groups of patients [4], including patients with CAD [11]. In CAD patients, some studies have tested relationships identical with some of the dimensions of HRQOL model [3,12,13] showing that neither impaired left ventricular ejection or ischemia, using non-invasive cardiovascular testing, were associated with physical function or general health perception [3,13]. Further, Gehi et al [12] did not find any association between self-reported angina pectoris and objective evidence of inducible ischemia in patients with known CAD. A recent study by Mathisen et al [14] showed reciprocal relationships between general health perception and overall QOL after coronary artery bypass surgery. In older women with heart disease, where arrhythmia, angina, myocardial infarction, congestive heart failure or valvular disease were included, Janz et al [15] found that overall QOL was significantly related to measures representing each of the dimensions suggested by Wilson and Cleary [5]. More specifically, cross-sectional analyses using linear regression models showed that general health perception explained more of the variation in QOL (38%) than any other category, while biological and physiological factors explained 13%. When considered jointly, all model variables explained 47% of the variation in overall QOL [15].

Although different studies have looked into several dimensions of HRQOL, it has not yet been fully evaluated in patients with CAD. For instance, anxiety and depression, which are common symptoms in these patients, have rarely been included in evaluating the associations between disease severity and dimensions of HRQOL. Höfer et al [10] did include anxiety and depression as individual characteristics that were supposed to shape the appraisal of health status in patients referred for angiographic evaluation of chest pain. They found that symptoms of depression and anxiety were the most important mediator variables in the process toward HRQOL. Using structural equation modelling, their results provide support for the proposed model by Wilson and Cleary. Also Ruo et al [3] found that depressive symptoms in patients with CAD were strongly associated with self-reported symptom burden, physical limitation, QOL and overall health. In addition, several studies have indicated that the way people cope with their perception of illness may influence their physical and psychological well-being [16,17]. To our knowledge no study has previously included use of coping strategies in evaluating associations between disease severity and HRQOL dimensions in CAD patients. Coping is claimed to be one of the core concept in medical and health psychology, and is strongly associated with the regulation of emotions throughout the stress period [18]. It is recognised that the way patients are coping with the stress and disability related to CAD, may effect subsequent adjustment and is of importance for their well-being [19,20].

By improving our understanding of the characteristics which are associated with symptoms, function, coping and well-being in CAD patients, the health care system might provide better therapy and care for the patients [1,3,5,21,22]. CAD is a chronic disease that has to be managed rather than cured. Therefore, knowledge about the relationships between objective disease factors and patients experience of its impact on daily life, might be relevant and useful in the communication with patients when planning treatment and rehabilitation [4].

Motivated by Wilson and Cleary's model [5], our overall aim was to investigate associations between disease severity and both mental and physical dimensions of HRQOL in patients admitted for elective coronary angiography. Our specific research questions were to explore the relation of disease severity with symptoms of angina, dyspnea, anxiety and depression, and how these factors relate to functioning, coping, perceived burden of living with angina pectoris, general health perception and overall QOL?

### Conceptual model

Wilson and Cleary have proposed a conceptual model, based on theory, clinical practice and research findings, to distinguish among conceptually distinct measures of HRQOL [5]. By this model they hypothesise associations between different levels of HRQOL and overall QOL. The model is divided into five levels 1) biological and physiological factors, 2) symptom status, 3) functional status, 4) general health perception and 5) overall QOL, and thereby integrates the biological and physiological factors with patients's subjective experiences of living with the disease.

Because emotional or psychological factors could be classified at different levels, Wilson and Cleary did not include these factors in their model. However, they argue that they may classify for example depression as a measure of symptom status, although some would argue that it could be classified as a biological or physiological factor, or as a measure of psychological function. The model also links characteristics of the individual and the environment [5].

**Figure 1:**
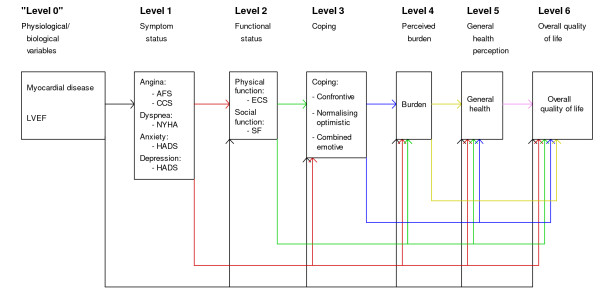
**A modified version of the Wilson & Cleary model**. LVEF: Left ventricular ejection fraction; AFS: Angina Frequency Scale; CCS: Canadian Cardiovascular Society classification; NYHA: New York Heart Association; HADS: Hospital Anxiety and Depression Scale; ECS: Exertional Capacity Scale; SF: Social Function; Coping: Confrontive coping, Normalising Optimistic Coping, Combined Emotive coping; Burden: Perception of living with angina pectoris.

Coping is not made explicit in the model developed by Wilson & Cleary. However, coping may be seen as any effort to manage or adapt to perceived external or internal demands [19]. Thereby, one may propose that coping is a mediator between functional status and the perception of burden in the HRQOL model by Wilson and Cleary [5]. According to Lazarus and Folkman [19], coping covers both problem-focused and emotion-focused coping. The first is aimed at changing the situation causing the distress and to relieve the perceived problem, while the second is aimed at changing the emotions caused by the stressful event. We therefore suggest that different coping strategies used by patients admitted for elective coronary angiography may have an impact on their perceived burden, general health perception and overall QOL. Figure 1 outlines the modified version of the Wilson and Cleary model used in the present study.

## Methods

### Design and subjects

The study has a cross-sectional design. Between August 2000 and February 2002, 1283 patients were consecutively admitted to elective coronary angiography at the Department of Heart Disease, Haukeland University Hospital, Bergen, Norway. At least 214 of the patients were not invited to participate due to capacity reasons. This means that on particular days or weeks with limited staff resources, usually caused by illness/sick leaves or by summer vacation, none of the patients were asked to participate. Among the remaining 1069 eligible patients, 753 patients (70%) responded and constitute the study population. Ethical recommendation was obtained from the Regional Committee of Medical Research Ethics, Norway. The participants delivered written informed consent after having received written information about the study.

### Clinical examination before angiography

All patients underwent a clinical examination before the angiography.

Before the clinical examination, the patients completed a questionnaire assessing prior history of heart disease and other illnesses, coronary risk factors, habitation status and educational level. During the consultation, they were asked to complete the questionnaires presented below, before they returned for angiography one to four days later.

### Measures

#### Physiological factors

Cardiac catheterisation was performed according to routine procedures. The presence of CAD was defined as a stenosis of at least 50% of the vessel lumen diameter in any of the main coronary arteries or their major side branches. The extent of CAD (0–3) was scored as the number of main vessels or side branches affected by stenoses [23]. Left ventricular ejection fraction (LVEF) was assessed by ventriculography.

#### Symptoms

Angina pectoris and dyspnoea were classified by the examining physician according to severity of symptoms by the Canadian Cardiovascular Society (CCS) [24] and New York Heart Association (NYHA) [25] classifications, respectively. The CCS classification consists of the following: Class 0: no angina, no limitations of physical activity by pain; Class I: ordinary physical activity does not cause angina, such as walking and climbing stairs; Class II: slight limitation of ordinary activity; Class III: marked limitation of ordinary physical activity; Class IV: inability to carry on any physical activity without discomfort – anginal syndrome may be present at rest [24]. The NYHA classification consists of the following: Class I: patients with cardiac disease but without resulting limitations of physical activity; Class II: patients with cardiac disease resulting in slight limitation of physical activity; Class III: patients with cardiac disease resulting in marked limitation of physical activity; Class IV: patients with cardiac disease resulting in an inability to carry on any physical activity without discomfort [25].

Symptoms of angina pectoris was also measured by self-report using the Anginal Frequency Scale (AFS) (2 items), one of the five subscales of the Seattle Angina Questionnaire (SAQ) [26], quantifying the number of angina episodes. AFS is transformed to a score of 0 to 100, where higher scores indicate better functioning. The SAQ is a valid and reliable disease-specific, self-administered instrument [27,28]. In the present study, internal consistency (Cronbach's alpha) for AFS was 0.77.

Anxiety and depression were assessed by self-report using the Hospital Anxiety and Depression Scale (HADS), which consists of seven items for anxiety (HADS-A) and seven for depression (HADS-D) [29]. Each item is scored from 0 (not present) to 3 (maximally present). Valid rating is defined as at least five completed items, and a summary score of at least eight is recommended to classify clinically relevant anxiety or depression [29]. The HADS takes only a few minutes to complete [30]. In the present study, internal consistency (Cronbach's alpha) for the HADS-A and HADS-D were 0.85 and 0.77, respectively.

#### Functional status

Self-reported functional status was assessed by the Exertional Capacity Scale (ECS) consisting of nine items measuring physical function, a subscale of the disease specific SAQ. Social function was measured by the Social Functioning scale (SF) consisting of two items, a subscale of the Short Form-36 (SF-36) [31]. All scores for both the ECS and SF were linearly transformed so that the lowest and highest possible scores were 0 and 100, respectively. Zero is the worst and 100 the best possible health status. The SF-36 is a well-validated and reliable questionnaire for many groups, including patients with CAD [32,33]. In the present study, internal consistency (Cronbach's alpha) was 0.87 for the ECS and 0.82 for the SF.

#### Coping

Coping was assessed by self-report using the Jalowiec Coping Scale (JCS, revised 60 item version) [34], using the Norwegian version translated by Wahl et al with the following three coping subscales identified therein based on 31 items [35]; 1) Confrontive problem solving subscale, 2) Normalising optimistic subscale, and 3) Combined emotive subscale. In a recent validation study [36], it was stated that this model may be used in this population with some caution. An alternative version of this model suggested by the validation study was therefore used in a sensitivity analysis, as described in statistical analysis. In the present study, internal consistency (Cronbach's alpha) was 0.83 for the Confrontive problem solving, 0.80 for the Normalising optimistic and 0.76 for the Combined emotive subscale.

#### Patients' perception of living with angina pectoris (perceived burden)

Patients' perception of living with angina pectoris (perceived burden) was assessed by self-report using a single-item; "Do you find it difficult to live with angina pectoris?", with six alternative responses: 1) Yes, I feel it is a daily burden; 2) Yes, I think about it a lot; 3) Yes, sometimes; 4) No, rarely; 5) No, I hardly ever think about it; 6) I feel exactly the same as people who do not suffer from angina pectoris [37].

#### General health perception

General health was assessed by self-report, using the General Health (GH) – five items, a subscale of the SF-36, see above. In the present study, internal consistency (Cronbach's alpha) was 0.69.

#### Overall QOL

Self-reported overall QOL was measured using a single question of overall satisfaction with life; "When you think about your life at the moment, would you say that you by and large are satisfied with life, or are you mostly dissatisfied?". It contains seven alternative responses: 1) Very satisfied; 2) Fairly satisfied; 3) Satisfied; 4) So-so; 5) Dissatisfied; 6) Fairly dissatisfied; 7) Very dissatisfied [37].

### Statistical analysis

In computing scale scores, missing substitution by the means of non missing items in the subscale was performed in accordance with the manual and as suggested in the literature when at least 50% of the questions were answered [31,38].

The model used is shown in Figure 1. Variables included in "Level 0" are independent variables and all variables in "Level 1" are dependent variables. The variables in "Level 0" and "Level 1" are independent variables for "Level 2", and the variables in "Level 0, 1 and 2" are independent for "Level 3", and so on. Thus, all variables in previous levels are included as independent variables for outcome variables on a specified level.

For all dependent variable at each model level a regression model by all independent variables at that level was fitted. For CCS (four categories) and NYHA ordinal logistic regression was used, while linear regression was used for all other analyses, including perceived burden of living with angina pectoris (level 4) and overall satisfaction with life (level 6) since these were 6- and 7-category ordinal variables with no substantial skewness. All models were investigated based on singly imputed data using the function transcan in Harrell's package Design [39], before they were finally fitted using multiply imputed data (Design function aregImpute with 10 imputations), with non-imputed versions of dependent variables used in all analyses. Transcan was also used to decide what continuous variables should be entered linearly or non-linearly (using splines with four knots) in the models. Single imputations used the independent variables in the regression in question, while multiple imputations were based on all variables. All imputations also used LVEF from ultrasound measurements in addition to the variables in the model. For each model a single preparatory test for all two-way interactions was performed, deleting nonlinear terms and a few interactions indicated as unstable from the testing procedure if necessary, for making the interaction test feasible. If interactions were indicated this was reported, but for lack of substantiated interaction hypotheses we did not include interactions in the models.

For the three coping dimensions, alternative definitions were used in a sensitivity analysis. Specifically, the three items from the other scales that load on the Confrontive problem solving scale in the modified model (Table 1) [36] are included in the alternative Confrontive problem solving scale, and similarly for items with 'cross loadings' on the Normalising optimistic and the Combined emotive scale. One item with negative cross loading was reversed before inclusion in the alternative Normalising optimistic scale. All analyses involving coping scales were repeated with these alternative definitions, and the results were compared with main analyses.

**Table 1:** Regression analyses at levels 3–6, sensitivity analysis using alternative definitions withthe cross-loadings of coping scales.

	**Co^a^**	**No^a^**	**Ce^a^**	**Burden^b^**	**GH^c^**	**QOL^d^**
CAD ^e^	-0.09	-0.01	0.10	0.16	1.73	0.07
AFS ^f^	-0.03	-0.05	-0.02	0.02; ***	0.03	-0.00
HADS-A ^g^	1.19; ***	0.80; **	1.57; ***	-0.07; ***	-0.54;°	0.07; ***
HADS-D ^h^	-0.43	-1.39; ***	0.69; **	0.00	-0.70; *	0.06; ***
CCS ^i^				***		
I vs. 0	0.65	0.63	1.33	-0.34; *	-1.91	-0.21
II vs. 0	-0.75	0.23	2.24	-0.53; ***	-2.62	-0.21;°
III vs. 0	-0.31	2.50	3.03	-0.49; **	-1.39	-0.24
NYHA ^j^						
II vs. 0–I	-0.62	1.31	0.07	0.16	-1.62	0.08
III-IV vs. 0–I	-3.02	-3.14	1.22	0.15	-4.44;°	0.02

ECS^k^	-0.06	-0.11; *	-0,06	0.02; ***	0.23; ***	0.00
SF^l^	-0.04	-0.01	-0.10; ***	0.00;°	0.10; **	-0.01; ***

Co^a^				0.01; **	0.11; *	-0.01; *
No^a^				-0.00	-0.01	-0.00
Ce^a^				-0.01; ***	-0.19; **	0.00

Burden^m^						***
5 vs. 6					-1.38	-0.28
4 vs. 6					-0.39	-0.51; **
3 vs. 6					0.15	-0.53; **
2 vs. 6					2.01	-0.64; **
1 vs. 6					2.20	-1.12; ***

Adjusted R^2^	0.13	0.09	0.45	0.48	0.40	0.43

Interactions ^t^	0.34	0.76	0.33	0.25	0.21	0.29

^q ^CAD: Coronary artery disease vs no CAD (after angiography)

^a ^AFS: Angina Frequency Scale (Seattle Angina Questionnaire), scale score 0 (worst) to 100 (best).

^b ^HADS-A: Anxiety (Hospital Anxiety and Depression Scale), scale score 0 (best) to 21 (worst).

^c ^HADS-D: Depression (Hospital Anxiety and Depression Scale), scale score 0 (best) to 21 (worst).

^d ^ECS: Exertional Capacity Scale (Seattle Angina Questionnaire), scale scores 0 (worst) to 100 (best).

^e ^SF: Social Function (SF-36), scale scores 0 (worst) to 100 (best).

^f ^Co: Confrontive coping, No: Normalising Optimistic and Ce: Combined Emotive coping. The three dimensions in Wahl et al's model [33] of the Jalowiec Coping Scale [32]

^g ^Burden: Perceived Burden- perception of living with angina pectoris, 1 (worst) to 6 (best).

^h ^GH: General Health (SF-36), scale scores 0 (worst) to 100 (best).

^i ^QOL: Overall quality of life, 1 (best) to 7 (worst).

^t ^All two-way interactions, overall p-value, feasible after a few simplifications if necessary.

°p ≤ 0.10; * p ≤ .05. ** p ≤ .01. *** p ≤ .001

For CCS and NYHA the validity of a unified ordinal logistic regression model was assessed by diagnostic plots as recommended by Harrell [39], together with an inspection of the validity of both a proportional odds (PO) and a continuation ratio (CR) model, including a formal test for the CR model [39]. If these assumptions were considered as unreasonable, separate logistic regression models were fitted. If this test was non-significant, a unified model was fitted by PO or CR as judged from the diagnostic plots. The regression analyses used the statistical program R [40], while SPSS version 15 (SPSS Inc, Chicago, IL, USA) was used for descriptive analyses. A p-value of < 0.05 was classified as statistically significant.

### Clinical relevance and regression relationships

Some of the statistically significant regression relationships may not be very strong. To judge this matter we used the following guidelines. For continuous variables measured on a 0–100 scale (including coping), we assume that a 5 point difference is of some, and a 10 point difference of substantial clinical relevance, if other information is not available [8,41]. For relationships between two variables on a 0–100 scale, a regression coefficient below 0.5 (5/10) in absolute value means that more than 10 points in the independent variable is needed to correspond to a minimally relevant difference of 5 points in the dependent variable, this is considered as a rather weak relationship. For the HADS scales, with minimum 0 and maximum 21, we similarly assume that about a one point difference is of some, and a two point difference is of substantial clinical relevance. A relationship involving a HADS score as independent variable is therefore considered weak if the regression coefficient is below 2.5 (5/2), and a relationship involving a HADS score as dependent variable is considered weak if the regression coefficient is below 0.1 (1/10). For burden (6 point scale) and overall QOL (7 point scale), a one point difference is considered as substantial. When these variables are dependent, regression coefficients of about 0.1 (0.5 for HADS scales) are considered as appropriate.

## Results

### Characteristics of the study population

Table 2 presents demographic and clinical characteristics of the 196 women and 557 men, admitted for elective coronary angiography. The mean (SD) age for women was 63 (10.4) years and for men 61.3 (10.1) years. Angiographic CAD was found in a majority (81%) of the patients, and was significantly more frequent in men. The mean value of the LVEF was 64.6 (12.0), and 12% of the participants had LVEF below 50%. A majority (82%) of the participants had angina pectoris and most of them were graded with CCS class II, and none was graded with class IV. Dyspnea was less frequent (34%), and mostly graded with NYHA class II.

**Table 2:** Demographic and clinical characteristics of study population

**Variables**	**N = 753**		
	
	**N**	**Mean (SD)**	**%**
Age		61.7 (10.2)	
Gender			
Women			26
Men			74
Living alone	723		16
Education	718		
Primary school			47
High school			33
>12 years/college/university			21
Smoking	735		
No-smoker			33
Ex-smoker			45
Current smoker			22
Non-cardiac diseases/other health complaints	538		89
Diabetes Type I or II	751		10
Body mass index (BMI) kg/m^2^	751	26.8 (4.2)	
CCS classification of angina ^a^	752		
Class 0 (no angina)			19
Class I			13
Class II			51
Class III			18
NYHA classification of dyspnea ^b^	750		
NYHA I (no dypnea)			66
NYHA II			26
NYHA III-IV			8
Coronary artery disease ^c^			
No			19
Yes			81
Left ventricular ejection fraction unit^d^	663	64.6 (12.0)	
HADS-anxiety	632	5.5 (4.0)	
HADS-depression	632	3.9 (3.3)	
Angina Frequency Scale (AFS)	682	62.7 (28.5)	
Exertional Capacity Scale (ECS)	698	66.2 (18.9)	
Social Function (SF)	725	74.6 (25.1)	
General Health (GH)	715	58.1 (19.4)	
Confrontive coping^e^	549	1.44 (0.61)	
Normalising optimistic coping^e^	582	2.17 (0.54)	
Combined emotive coping^e^	590	0.89 (0.57)	
Perception of living with angina pectoris	612	3.9 (1.4)	
Overall quality of life	624	3.2 (1.3)	

^a ^Canadian Cardiovascular Society classification

^b ^New York Heart Association

^c ^Angiographic diameter stenosis of at least 50% in at least one of the main coronary arteries or their major side branches

^d ^Left ventriculography was performed in 88% of the patients

^e ^Alternative mean (SD) scores for coping using a 0–100 scale: Confrontive coping: 47.9 (20.4), Normalising optimistic: 72.4 (18.1) and Combined emotive coping: 29.5 (18.9).

The mean value of symptoms of angina pectoris measured by AFS was 62.7 (28.5). HADS scores of 8 or more, indicating anxiety, were found in 26% of the patients, while HADS-depression scores of at least 8, indicating depression were found in 15% of the participants.

### Regression analyses

Nonlinearity was indicated for LVEF and body mass index and for General Health at level 6. All other continuous independent variables were entered linearly into the models. The results for the linear and logistic regressions are reported in Table 3 and 4, respectively.

**Table 3:** Regression analyses for angina (Angina Frequency Scale), anxiety and depression (Hospital Anxiety and Depression Scale), functioning (Exertional Capacity Scale and Social Function), coping (Confrontive coping, Normalising Optimistic coping, Combined Emotive coping scales), perceived burden, general health and overall quality of life.

	**AFS^a^**	**HADS-A^b^**	**HADS-D^c^**	**ECS^d^**	**SF^e^**	**Co^f^**	**No^f^**	**Ce^f^**	**Burden^g^**	**GH^h^**	**QOL^i^**
CAD^q^	-9.49;**	-0.36	0.47	-0.50	-1.13	0.42	-0.49	-0.16	0.16	1.74	0.08

AFS^a^				0.23; ***	0.14; ***	-0.03	-0.06;°	-0.02	0.02; ***	0.03	-0.00
HADS-A ^b^				-0.22	-1.91; ***	1.32; ***	0.79; **	1.75; ***	-0.07; ***	-0.59;*	0.06; ***
HADS-D ^c^				-1.09; ***	-2.42; ***	-0.38	-1.41; ***	1.40; ***	-0.00	-0.74; *	0.06; **
CCS ^j^				***					***		
I vs. 0				-3.16	-0.04	1.99	0.89	-0.56	-0.37; **	-2.23	-0.19
II vs. 0				-2.48	3.36	-0.72	0.08	0.88	-0.55; ***	-2.77	-0.21;°
III vs. 0				-9.09; ***	0.42	-0.58	2.60	2.72	-0.50; **	-1.52	-0.24
NYHA ^k^				***	*						
II vs. 0–I				-3.55; **	-1.16	-0.40	0.95	-0.41	0.16	-1.70	0.08
III-IV vs. 0–I				-8,01; ***	-8.17; ***	-2.40	-3.64	1.44	0.11	-4.75;°	0.03

ECS^d^						-0.07	-0.12; *	-0,00	0.02; ***	0.23; ***	0.00
SF ^e^						-0.05	0.01	-0.11; ***	0.00; *	0.11; **	-0.01; ***

Co^f^									0.01; *	0.07;°	-0.01; *
No^f^									-0.01; **	-0.03;	-0.00
Ce^f^									-0.01°	-0.10;°	0.01;°

Burden^g^											***
5 vs. 6										-1.19	-0.29
4 vs. 6										-0.07	-0.52; **
3 vs. 6										0.62	-0.54; **
2 vs. 6										2.56	-0.64; **
1 vs. 6										3.04	-1.14; ***

Adjusted R^2^	0.05	0.12	0.06	0.42	0.39	0.15	0.09	0.51	0.48	0.39	0.43

Interactions ^t^	0.87	0.30	0.37	0.19	0.067	0.37	0.75	0.023	0.38	0.30	0.44

Analyses adjusted for gender, age, education, cohabitation, smoking, body mass index (BMI), diabetes and co morbidity. Regression coefficients; p-values are presented.

^q ^CAD: Coronary artery disease vs no CAD (after angiography)

^a ^AFS: Angina Frequency Scale (Seattle Angina Questionnaire), scale score 0 (worst) to 100 (best).

^b ^HADS-A: Anxiety (Hospital Anxiety and Depression Scale), scale score 0 (best) to 21 (worst).

^c ^HADS-D: Depression (Hospital Anxiety and Depression Scale), scale score 0 (best) to 21 (worst).

^d ^ECS: Exertional Capacity Scale (Seattle Angina Questionnaire), scale scores 0 (worst) to 100 (best).

^e ^SF: Social Function (SF-36), scale scores 0 (worst) to 100 (best).

^f ^Co: Confrontive coping, No: Normalising Optimistic and Ce: Combined Emotive coping. The three dimensions in Wahl et al's model [33] of the Jalowiec Coping Scale [32]

^g ^Burden: Perceived Burden- perception of living with angina pectoris, 1 (worst) to 6 (best).

^h ^GH: General Health (SF-36), scale scores 0 (worst) to 100 (best).

^i ^QOL: Overall quality of life, 1 (best) to 7 (worst).

^j ^CCS: Canadian Cardiovascular Society Angina Classification, 0 (no angina) to IV (worst, not present in our data).

^k ^NYHA: New York Hear Association Dyspnoea Classification, 0 (no dyspnoea) to IV (worst). 0 and I, and III and IV, collapsed in our data due to small numbers.

^t ^All two-way interactions, overall p-value. Feasible after a few simplifications if necessary.

°p ≤ 0.10; * p ≤ .05. ** p ≤ .01. *** p ≤ .001

**Table 4:** Ordinal logistic regression for angina pectoris (CCS) (proportional odds models), logistic regression for dyspnea (NYHA).

	**CCS^c^**	**NYHA^d ^II-IV vs. NYHA 0–I**	**NYHA^d ^III-IV vs. NYHA II**
CAD ^a^	2.98; ***	0.42; ***	2.40;°
LVEF ^b^	**	***	
30 vs. 20	1.56	0.49	0.61
50 vs. 40	1.52	0.51	0.64
70 vs. 60	1.02	1.12	1.10

Interactions ^t^	0.56	0.89	

Odds ratios; p-values are presented.

^a ^CAD: CAD vs no CAD (after angiography).

^b ^LVEF: Left ventricular ejection fraction. Nonlinear relationships entered, differences for selected LVEF intervals are presented.

Significantly associated to CCS (**), and to NYHA (II-IV vs. 0–I, ***). Nonlinearity: Significant for NYHA (II-IV vs. 0–I, **).

^c ^CCS: Canadian Cardiovascular Society Angina Classification, 0 (no angina) to IV (worst, not present in our data).

^d ^NYHA: New York Hear Association Dyspnoea Classification, 0 (no dyspnoea) to IV (worst). 0 and I, and III and IV, collapsed in our data due to small numbers

^t ^All two-way interactions, overall p-value. Not feasible for NYHA, feasible after a few simplifications if necessary

°p ≤ 0.10; * p ≤ .05. ** p ≤ .01. *** p ≤ .001

### Determinants of symptoms

**Figure 2:**
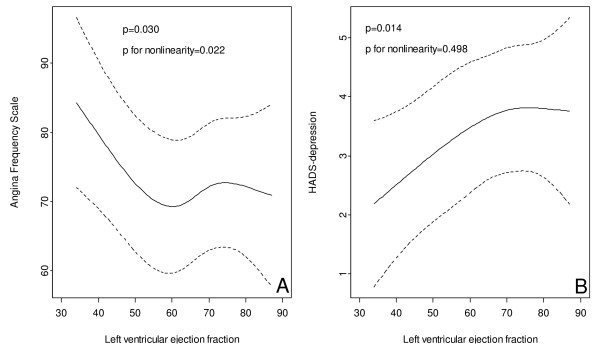
A: Association between left ventricular ejection fraction and angina (Angina Frequency Scale). B: Association between left ventricular ejection fraction and depression (HADS).

We found significant relationships between biological variables and the patient's perceived symptoms (Table 3). As shown in this table, we found a significant and appreciable association between angiographically confirmed CAD and self-reported symptoms of angina pectoris (AFS) (coefficients: -9.49, p = 0.002). As shown in figure 2(A), LVEF was significantly (p = 0.030) related to self-reported angina pectoris (AFS), with a substantially less angina symptoms with decreasing LVEF values below about 50–60%. Also angina (CCS) (OR 2.98, p < 0.001) and dyspnea (NYHA) (OR 0.45, p < 0.001), as graded by the examining physician, were significantly related to the presence of CAD (Table 4). CAD had a strong and positive relationship with CCS, and a negative relationship with dyspnea (NYHA II-IV). CCS symptoms increased with increasing LVEF (p = 0.002), and NYHA symptoms increased with decreasing LVEF, below about 50–60%. Figure 2(B), shows that symptoms of depression were positively related to LVEF (p = 0.014), possible less so for LVEF values above about 60–70%.

### Determinants of functional status

As shown in Table 3, both angina pectoris (AFS, coefficient: 0.23, p < 0.001 and CCS, p < 0.001) and dyspnea (NYHA, p < 0.001) were significantly related to impaired physical function (ECS). Physical function was substantially lower in patients with the most severe symptom of angina pectoris (CCS, coefficient: -9.09, p < 0.001), and dyspnea (NYHA, coefficient: -8.01, p < 0.001), while the relationship between AFS and ECS was significant, but not particularly strong (coefficient: 0.23, p < 0.001). Symptom of depression was significantly, although rather weakly, related to impaired physical function (coefficient: -1.09, p < 0.001). There was a positive, but weak, relationship between self-reported angina pectoris (AFS) and social function (coefficient: 0.14, p < 0.001). Social function was appreciably lower in patients with severe dyspnea (coefficient: -8.17, p < 0.001). Social function was somewhat lower in patients with more symptoms of anxiety (coefficient: -1.91, p < 0.001) and depression (coefficient: -2.42, p < 0.001).

### Determinants of coping

There was a significant, but rather weak, relationship between anxiety and more use of confrontive coping (coefficient: 1.32, p < 0.001), normalising optimistic (coefficient: 0.79, p = 0.002) and combined emotive coping (coefficient: 1.75, p < 0.001) (Table 3). Similarly, there were somewhat weak but statistically significant relationships between symptoms of depression and less use of normalising optimistic coping (coefficient: -1.41, <0.001), and more use of combined emotive coping (coefficient: 1.40, p < 0.001). There were also weak but statistically significant relationships between physical function (ECS) and less use of normalising optimistic coping (coefficient: -0.12, p = 0.037), and between social function and less use of combined emotive coping (coefficient: -0.11, p < 0.001). The relationships using the alternative coping scale specifications using cross loadings (Table 1) were similar, but with an even weaker relationship between symptoms of depression and combined emotive coping.

### Determinants of perception of living with angina pectoris (perceived burden)

Symptoms of angina pectoris (AFS) (coefficient: 0.02, p < 0.001) and anxiety (coefficient: -0.07, p < 0.001), and use of normalising optimistic coping (coefficient: -0.01, p = 0.032), were significantly related to more burden, while physical function (ECS) and use of confrontive coping were significantly related to less burden. These relationships were weak. Patients with angina pectoris perceived more burden. The relationships using the alternative coping scale specifications using cross loadings (Table 1) were similar. However, the relationship with normalising optimistic coping was of similar magnitude, but not significant.

### Determinants of general health

General health was negatively related to symptoms of anxiety (coefficients: -0.59, p = 0.037) and depression (coefficient: -0.74, p = 0.036) and positively related to physical (ECS) (coefficient: 0.23, p < 0.001) and social function (coefficient: 0.11, p = 0.001). All these relationships were weak (Table 3). The relationships were similar using the alternative coping scale specifications using cross loadings (Table 1). Here, in addition, general health was positively related to use of confrontive coping and negatively related to normalising optimistic coping. These relationships were weak, but somewhat stronger than in the corresponding relationships presented in Table 3.

### Determinants of overall QOL

Better overall QOL was significantly related to less symptoms of anxiety (coefficient: 0.06, p < 0.001) and depression (coefficient: 0.06, p = 0.001), these relationships were weak. Also, overall QOL was significantly and negatively related to social function (coefficient: -0.08, p < 0.001) and use of confrontive coping (coefficient: -0.01, p = 0.017). Overall QOL was lower for patients with more perceived burden of living with angina pectoris (coefficient: -1.14, p < 0.001). These relationships were similar using the alternative coping scale specifications using cross loadings (Table 1), except the relationships with social function and confrontive coping that were appreciably weaker.

## Discussion

In patients undergoing elective cardiac catheterisation, we examined relationships between coronary artery disease severity and several measures of HRQOL, and overall QOL. This was motivated by a theoretical model by Wilson and Cleary. Furthermore, to our knowledge this is the first study that has included use of coping strategies and perceived burden in evaluating associations between disease severity and HRQOL dimensions. Our findings support their proposed model to a large extent.

We found that patients with angiographically evident CAD had more angina pectoris and less dyspnea, which are the classic symptoms of ischemic heart disease, than patients without significant narrowing coronary arteries. This gives support to the proposed relationship of biological and physiological variables, with symptoms, and is in accordance with results reported by Höfer et al [10], who found significant relationships between diseased vessels and angina pectoris in patients with angiographically documented CAD. In contrast, Gehi et al [12] found no association between objective evidence of ischemia in patients with known CAD and self-reported angina pectoris, measured by the AFS. In the study by Gehi et al [12], noninvasive imaging for the evaluation of CAD was performed by stress echocardiography, which evaluates the hemodynamic sequelae rather than the anatomical extent of CAD per se. Although the test result is usually significantly related with the prevalence of CAD at angiography [42], these differences in cardiac endpoints as well as in patient characteristics reflecting different recruitments regimens and institutional referral patterns, probably explain the discrepant results.

Among patients with CAD, Ruo et al [3] reported that impaired LVEF measured by echocardiography and inducible ischemia on stress echocardiography were not associated with symptom burden of angina pectoris, measured by the AFS. In our study, reduced angina frequency was found in patients with impaired ventricular function. The reason may be lack of myocardial viability after previous infarction or that people with worse LVEF do not exert themselves enough to have angina symptoms. In addition, patients with severe dysfunction from ischemic cause, initially have less angina pectoris due to severely damaged myocardium.

Because anxiety and depression are frequent symptoms in patients with CAD [3,43,44], we also in contrast to Wilson and Cleary, included these symptoms in our model. Whereas anxiety was neither associated with the extent of CAD nor with LVEF, depression was significantly related to LVEF with less depressive symptoms found in patients with impaired ventricular function. Thus in the present population, depression is not likely to be secondary to impaired ventricular function. Indeed, previous investigations have shown that depression and impaired LVEF are independently associated with a poor prognosis in CAD patients, and assessment of the relationship between depression and LVEF is therefore assumed to be of great importance [44,45]. There are few prior data on this relationship [45]. Our result of less depression in patients with LVEF dysfunction is in contrast to results reported in patients hospitalised for acute myocardial infarction [45]. Lack of association between LVEF and depression has previously been reported by Ruo et al [3] in a large sample of patients with documented CAD. However, in contrast to our study, they found strong relationship between depressive symptoms and self-reported HRQOL. The design of our study investigating patients referred for elective cardiac catheterisation is not quite similar to the study by Ruo et al [3] and may influence the different results. We used a modified version of the Wilson and Cleary model adhering to the suggested relationship between variables. We also included anxiety and coping and different measures are used for some variables, including depression and overall quality of life.

We found no significant relationship between LVEF and any of the other HRQOL variables. The absence of associations between LVEF and both physical function and general health, has also been reported by Mattera et al [13]. It has been argued that generally there is a weak relation between the severity of CAD as evaluated by coronary angiography, and patient-reported health status [22]. In accordance with this, our results showed that the extent of CAD was not associated with disease specific and self-reported physical function.

Physical function was significantly related to angina pectoris and dyspnea. Impaired physical function was more clearly uncovered in patients with the most severe angina, classified by the CCS, which is in accordance with a previous report [11], whereas a weaker relation was observed when angina pectoris was measured by the AFS. Social function was weakly associated with angina pectoris, while the relationship with dyspnea was stronger and probably of clinical importance.

Although depression was significantly related to impaired physical function, and anxiety to decreased social function, these associations were weak and hardly of clinical importance. A weak association between depression and physical limitation has also been reported by Sullivan et al [11], but is in contrast to previous research, where a strong relationship has been reported in patients with CAD [3,10,41]. A possible explanation for the contrasting results might be due to differences in cardiac population or questionnaires used for the assessment of depression.

With regard to coping, the results showed that the use of confrontive coping strategies was related to less perceived burden and better overall QOL. However, most of the other associations to the different coping strategies were weak. Emotion focused coping refers to thoughts and behaviour that an individual uses to regulate distress, while problem-focused coping is aimed at managing the problem causing distress [46]. Confrontive coping might be seen as a problem-focused strategy to change the situation causing distress. By using confrontive coping, the person tries to find out more about the problem or learn more to deal with the problem and so on. Greater control is associated with higher levels of problem-focused coping [46]. Emotion-focused coping has been associated with higher levels of distress [18,47]. However, coping is embedded in a complex, dynamic contextual process and therefore the interpretations of associations are difficult, especially when using a cross-sectional design. According to Folkman and Moskowitz (2004), coping has been found to be strongly associated with the regulation of emotion, such as distress [18]. Certain kind of escapist coping strategies are consistently associated with poor mental health outcomes, while other kind of coping, such as seeking social support or instrumental problem-focused forms of coping, are sometimes associated with negative outcomes, sometimes with positive ones, and sometimes with neither, usually depending on characteristics of appraisal stressful encounter.

The main goal of clinical care is to improve patient outcome. Partly due to an aging population of cardiac patients, therapeutic efforts also increasingly focus on improving patients functioning and wellbeing. Therefore, identification and understanding the relationships between different HRQOL factors are of great importance [5]. Further, patients self-report is essential in addition to the investigations by the physicians [22]. Wilson and Cleary (1995) included biological and physiological data and patients-reported symptoms in their model, but assert that traditionally it has not been included in conceptualisations of HRQOL. Although they recognise the importance of including emotional and psychological factors, they preferred to avoid it because of its complexity and the possibility that its causal relationship might go in both direction [5]. We included all these factors, and found that 43% of the variance of overall QOL was explained by this model.

Strengths of our study include the large sample size of cardiac patients. All questionnaires were completed before catheterization and responses were therefore not influenced by the results of the catheterization procedure. The investigation also incorporated several disease-specific instruments. A limitation of our study is the cross-sectional design, which highlights associations and not causality. The relatively low number of women did not allow us to study gender specific associations in detail. The stop in recruitment for shorter periods was not characterized by a systematic pattern and is unlikely to have caused substantial selection bias. Our sample was taken from a geographical region with an almost homogeneous caucasian population. We therefore cannot generalise to population with other ethnical compositions. It is also a limitation that the 60 item JCS has not been subject to extensive psychometric testing in previous literature. Our sensitivity analysis was therefore based on a single psychometric evaluation in the same patient sample.

## Conclusion

We observe distinct associations between classical cardiac and psychological symptoms in patients with suspected CAD, with physical and social function. Use of the confrontive coping strategy is related to less perceived burden and better overall QOL in these patients. Our data support the model suggested by Wilson and Cleary.

## Competing interests

The authors declare that they have no competing interests.

## Authors' contributions

BU contributed to all parts of this study by data collection, planning and designing the study, statistical analysis and in drafting the manuscript, ON coordinated the study at the hospital and participated in data collection, and in drafting the manuscript, BRH and AKW participated in planning and designing the study, and drafting the manuscript, TW-L participated in designing the study, performed the statistical analysis and participated in drafting the manuscript. All authors read and approved the final manuscript.
